# An Underestimated Toxicity Radiation-Induced Hypothyroidism in Patients Multimodally Treated for Breast Cancer

**DOI:** 10.3390/jcm10235503

**Published:** 2021-11-24

**Authors:** Camil Ciprian Mireștean, Roxana Irina Iancu, Dragoș Petru Teodor Iancu

**Affiliations:** 1 Department of Oncology and Radiotherapy, University of Medicine and Pharmacy Craiova, 200349 Craiova, Romania; mc3313@yahoo.com; 2 Department of Surgery, Railways Clinical Hospital Iasi, 700506 Iași, Romania; 3 Oral Pathology Department, Faculty of Dental Medicine, “Gr. T. Popa” University of Medicine and Pharmacy, 700115 Iași, Romania; 4 Department of Clinical Laboratory, “St. Spiridon” Emergency Universitary Hospital, 700111 Iași, Romania; 5 Oncology and Radiotherapy Department, Faculty of Medicine, “Gr. T. Popa” University of Medicine and Pharmacy, 700115 Iași, Romania; dt_iancu@yahoo.com; 6 Department of Radiation Oncology, Regional Institute of Oncology, 700483 Iași, Romania

**Keywords:** hypothyroidism, breast cancer, radiotherapy, supraclavicular irradiation

## Abstract

Radiation therapy is part of the therapeutic arsenal for breast cancer, whether it is adjuvant treatment after lumpectomy or radical mastectomy, or it is used as a palliative option in the case of metastatic or recurrent disease. Significant advances in diagnostic and therapeutic stratification of breast cancers have significantly prolonged survival, even in the metastatic stage. Exposure of patients during the course of the disease in a multidisciplinary therapeutic approach including chemotherapy, hormone therapy, targeted anti-HER therapies or CDK4/6 inhibitors had led to improved survival but with the price of additional toxicity. Among them, hypothyroidism is a well-known consequence of external radiation therapy, especially in the case of cervical region irradiation, including supraclavicular and infra-clavicular nodal levels. In this situation, the thyroid gland is considered as an organ at risk (OAR) and receives a significant dose of radiation. Subclinical hypothyroidism is a common endocrine disorder characterized by elevated TSH levels with normal levels of FT4 (free T4) and FT3 (free T3), and as a late effect, primary hypothyroidism is one of the late effects that significantly affects the quality of life for patients with breast cancer receiving multimodal treatment. Hypothyroidism has a significant impact on quality of life, most often occurring as late clinical toxicity, secondary to thyroid irradiation at doses between 30 and 70 Gy. Dose-volume parameters of irradiation, gland function at the beginning of the treatment and associated systemic therapies may be factors that alter thyroid radio-sensitivity and affect thyroid gland tolerance. In the case of head and neck tumor pathology, in which doses of >50 Gy are routinely used, the thyroid gland is generally considered as an OAR, the rate of radio-induced hypothyroidism being estimated at rates of between 20% and 52%. For breast cancer, the thyroid is often neglected in terms of dosimetry protection, the rate of late dysfunction being 6–21%.

## 1. Introduction

Radiation therapy is part of the therapeutic arsenal of breast cancer, whether it is used as an adjuvant treatment after lumpectomy or radical mastectomy, or as a palliative option in the case of metastatic or recurrent disease. Significant advances in diagnostic and therapeutic stratification of breast cancers have significantly prolonged survival, even in the metastatic stage. Exposure of patients during the course of the disease in a multidisciplinary therapeutic approach, including chemotherapy, hormone therapy, targeted anti-HER therapies or CDK4/6 inhibitors has led to improved survival with the price of additional toxicity. Among them, hypothyroidism is a well-known consequence of external radiation therapy, especially in the case of irradiation of the cervical region, including supraclavicular and infra-clavicular nodal levels. In this situation, the thyroid gland is considered as an organ at risk (OAR) and receives a significant dose of radiation. [1,2].

## 2. Radiation-Induced Thyroid Toxicity—From Pathophysiology to Clinical Symptoms

The toxic effect of radiation on the thyroid is classically produced by small vessels’ destruction and, to a lesser extent, by direct damage to the follicles, to a secondary, indirectly damage among vascular networks. These lesions are generally responsible for late and irreversible side effects, while acute and sub-acute lesions are caused by follicular damage. The latency period between the time of irradiation and the pathophysiological changes responsible for thyroid toxicity is well known, without a clear explanation of this latency. Among patients with occult hypothyroidism, one quarter will progress to clinical disease. Radiation-induced hypothyroidism is reported in 15–50% of patients, with symptoms occurring 2–4 years after treatment. Clinical hypothyroidism is considered as clinical disease when there is a high level of thyrotropin with low level of thyroxine (T4) and subclinical with a high level of thyrotropin and normal level of T4 [3,4,5,6]. Defined more than 50 years ago by Jefferies, occult hypothyroidism is characterized by vague and nonspecific complaints, few or absent clinical signs and specific laboratory values. Moreover, the response to treatment can be inconsistent or variable [7]. Most of the data on the involvement of radiation therapy in thyroid gland toxicity and, more precisely, the dose-volume-toxicity correlation comes from studies including patients with lymphoma and head and neck cancers [8,9].

Bhandore and collaborators mention a 3.1-year latency of hypothyroidism in head and neck cancer patients, considering that the risk is dependent on the radiation dose received by the thyroid gland. The authors report a rate of clinical and occult hypothyroidism of 33% and 29%, respectively, analyzing many cases, including 312 of head and neck cancer; however, the highest rate of hypothyroidism is reported by Mercado et al. (67% at 8 years after the irradiation treatment) in head and neck cancers. As demonstrated by Sinard and colleagues analyzing data from 198 patients with head and neck cancer, including patients with total laryngectomy followed by radiotherapy, the rate of hypothyroidism is only 15% at 12 months after treatment, with surgery being an additional factor risk. This study conceptually supports the delayed hypothyroidism induced by irradiation. Radiotherapy of the neck region is considered a major risk factor of thyroid toxicity, and neck dissection is also associated with an increased risk [5,6,8,10,11].

Hyperthyroidism is reported to be much less common, with an incidence of 1.7% in Hodgkin’s lymphoma patients treated with radiation therapy. Thyroiditis is rare as a complication of irradiation, but even if it is reduced as a side effect, the rate of thyroiditis is double in patients who have been treated with radiation therapy as part of multimodal treatment. In a cohort of 1787, 4 cases of Hashimoto’s tyroidites and 4 cases of “silent” tyreotoxicosis were reported, most of them being diagnosed in the first 2 years after Hodkin’s lymphomas radiotherapy of neck region. Illes and colleagues considered that in the case of Hodkin’s lymphoma, some of hypothyroidism may be related to thyroiditis, due to the increased immune reactivity associated with Hodgkin’s disease [5,7,8,9,10,11,12,13,14].

## 3. Thyroid Gland as an OAR—Dose-Volume-Toxicity Correlation

The risk of radio-induced hypothyroidism varies, involving factors related to the patient, the histological type, clinical stage of cancer and treatment administered. Older and younger age, female gender, Caucasian race and smaller thyroid gland volume are factors associated with an increased risk of late radio-induced hypothyroidism. Partial or total resection and neck dissection are also associated with an increased risk of hypothyroidism [15,16].

The radiation dose received by the thyroid gland is shown to be a predictor of late thyroid toxicity. Dose-response variation of irradiation-induced hypothyroidism is demonstrated, a 20-year assessment of treatment identifies a 20% risk associated with an average dose received by the thyroid gland <35 Gy. The risk increases to 30% for doses between 35 Gy and 44.9 Gy and reaches 50% if the thyroid is irradiated with doses >45 Gy. The risk is higher if childhood cancer patients have received cranial irradiation and neck irradiation. Thus, a dose of >20 Gy received by the pituitary gland associated with a dose of >10 Gy received by the thyroid gland is associated with hypothyroidism in acute lymphoblastic leukemia (ALL) children survivors who received craniospinal irradiation [17,18]. If there is a consensus that doses of >45 Gy are associated with an increased risk of hypothyroidism, the increase in risk at higher doses is not proportional to the dose received by the thyroid gland. Thus, Postner and colleagues demonstrate that the risk of hypothyroidism at a dose of >65 Gy is not higher than in the case of thyroid irradiation with doses between 45 and 65 Gy [3,5,19,20,21,22].

The development of new radiotherapy techniques with the possibility to delineate target volumes and organs at risk (OARs), identified and reconstructed from computer tomography (CT) simulation, have led necessity of dose-volume recommendations based on studies that have shown dose and volume correlation with hypothyroidism. In 2010, Quantitative Analyzes of Normal Tissue Effects in the Clinic (QUANTEC) included dose-volume-outcomes for most radiosensitive organs, but the thyroid gland was not included as an OAR in these guidelines [22]. The identification of dose-volume-effect correlations with the introduction of new radiotherapy techniques allows the protection of radiosensitive organs in order to limit adverse effects. The delimitation and three-dimensional reconstruction, together with computerized treatment planning system, allows the evaluation of the doses received by these radiosensitive organs and their sub-volumes. Modern radiotherapy techniques (3D-CRT, IMRT, VMAT) promise the irradiation of highly conformal target tumor volumes by using the multi-leaf collimator (MLC) and the three-dimensional reconstruction of both tumor volumes and the organs at risk. In the development of toxicities, not only the dose received by the whole organ was demonstrated as a predictor, but also the doses received by sub-volumes from different OARs [23,24,25]. For example, V30, one of the dosimetric parameter associated with the risk of hypothyroidism, is the volume percentage that receives a dose of at least 30 Gy [22,25].

With the implementation of new radiotherapy techniques, especially of IMRT irradiation, dose-volume constraints have been refined for all OARs including for the thyroid gland. Regarding the radiation-induced hypothyroidism, V30 has been identified as a predictor, with a 60% value correlated with a lower risk hypothyroidism. A V30 of 78% and >7 cm^3^ were associated with a higher rate of radiation-induced hypothyroidism. A V45 value of <50% and the sparing at least 5 cm^3^ of thyroid gland to receive lesser than 45 Gy, but also a 45 Gy mean dose for the thyroid gland of are the most common recommended dose-volume constraints in order to reduce the risk of hypothyroidism in radio-treated patients in the neck region [25,26,27,28]. After analysis of dose-volume-toxicity correlations on 345 cases of nasopharyngeal cancer treated by IMRT technique, with a median follow-up of 45.2 months, Huang and collaborators proposed V25 Gy ≤ 60%, V35 Gy ≤ 5 5%, and V45 Gy ≤ 45% for thyroid, as recommended constraints [29].

## 4. Breast Cancer—Major Diagnostic and Treatment Advances, New Toxicities

Breast cancer is the most common cancer in women worldwide, with an increasing incidence, especially in industrialized countries. Due to advances in diagnosis and therapy, life expectancy has increased considerably in the last 10–15 years. Screening programs have significantly contributed to early detection with maximum curative potential. Even it is discovered at an advanced stage, the multidisciplinary approach and the stratification of the disease according to the molecular subtypes (that benefit from specific therapeutic targets) has significantly improved the prognosis. The relative survival at 5 and 10 years is 88% and 77%, respectively, compared to 64% relative survival at 5 years from other cancers. Younger and older age, tumor size and skin involvement, histological type of invasive papillary, classic lobular and medullary and inflammatory breast, lympho-vascular invasion (LVI) are just some well-known negative prognostic factors. Moreover, the size of the tumor is associated with a larger number of positive nodes, thus indirectly influencing the prognosis. Nodal invasion is a significant prognostic factor, patients with positive nodes having a 4–8 times higher mortality rate than those without node involvement. Further, a number of 1–3 invaded nodes is associated with a 70% lower risk of death than a number >10 positive nodes. Distant metastases are associated with an unfavorable prognosis at 10 years, with survival estimated at 3.4%. These prognostic factors were combined into two main categories: the TNM system, which includes tumor size, nodal invasion status and distant metastases, and the Nottingham Prognostic Index (NPI) including tumor size, tumor histological grade and nodal status. Both classification systems have a significant and proven prognostic value [30,31,32,33].

A decisive contribution to the improvement of the prognosis of these patients is brought by the stratification of the therapy and the diversification of the therapeutic arsenal according not only to the TNM stage of the disease but also to the modern classifications including molecular particularities. The classification of breast cancer into four molecular and prognostic categories (Luminal A, Luminal B Her2 + and triple negative), is based on the status of estrogen receptors (ER), progesterone (PR), the status of HER2 and ki 67 amplification, a protein associated with tumor cell proliferation and growth. We will briefly present the diverse range of systemic therapies used in breast cancer without being the subject of this article [34,35,36]. Estrogen (ER) and progesterone (PR) hormone receptors are known as predictive and prognostic biomarkers for breast cancer; because of the Tamoxifen therapy benefit, ER+ patients have a better prognosis than ER- patients. Overexpression of the human epidermal growth factor type2 (HER2) receptor is associated with an unfavorable prognosis, with 10-year survival rate being 15% lower than in patients not expressing HER2. The association of HER2 with p53 gene abnormalities and high mitotic index explains the unfavorable prognosis associated with HER2+ [31,37,38,39]. It is estimated that 20–30% of breast cancer cases express HER2. Transtuzumab, Pertuzumab and Trastuzumab emtansine (T-DM1) are anti-HER2 therapies that improve the therapeutic outcomes of these patients in adjuvant and metastatic settings. One category that should be remembered for the potential for unfavorable prognosis is that of triple negative breast cancers (TNBC) that do not express estrogen and progesterone receptors or HER2 amplification. In this case, chemotherapy is the cornerstone of systemic therapy. New therapeutic options for metastatic disease including the tyrosine kinase inhibitor (TKI) Lapatinib and TDM-1 are active in brain metastatic HER2 + breast cancer, these pharmacological advances in systemic therapies bringing considerable benefits to metastatic disease as well [31,38,39,40,41]. Moreover, cyclin-dependent kinase inhibitor (Cdk4/6), Palbociclib, Ribociclib, Abemaciclib in combination with aromatase inhibitors (AI) demonstrated benefit in overall survival (OS) on subgroups of patients, including those with visceral metastases and resistance to endocrine initial therapy [42,43].

Some of these therapies have already been shown to interfere with thyroid function, but the hypothyroidism induced by systemic therapies administered in breast cancer is little known. By modulating Thyroxine-binding globulin (TBG) but also by acting on the synthesis and secretion of hormones in the thyroid gland, treatment with Tamoxifen is considered an additional risk factor for hypothyroidism in breast cancer patients. Gordon et al. demonstrate an increase in serum T4 concentrations after the start of Tamoxifen therapy in breast cancer patients, but this phenomenon is not seen in healthy volunteers who have received this treatment [44,45]. A case of hypothyroidism has also been reported in a 65-year-old patient treated with third-generation aromatase inhibitor exemestane 25 mg/day for 2 months. Rivas et al. incriminates a number of agents such as alkylating agents and taxanes used in breast cancers as contributors to the decrease in thyroid volume by 14–17%, thus being, indirectly, a cause of hypothyroidism and toxicity associated with irradiation [46]. Cyclophosphamide, ifosfamide and alkylating agents could increase T4 and reverse triiodothyronine (rT3) with a decrease in TSH unassociated with modified T3, thyroglobulin (Tg) and TBG, the hypothesis being based on release of thyroid hormone from other cellular deposits (possibly hepatic). Administration of docetaxel, doxorubicin, and cyclophosphamide have been associated with decreased serum T4 and increased TSH [47,48,49].

Adjuvant breast radiotherapy is essential not only in reducing the risk of loco-regional failures after tumorectomy/lumpectomy in cases of early breast cancer, but also in case of advanced breast tumors that benefit from chest wall irradiation after radical mastectomy. A meta-analysis, including 10,801 breast cancer patients, evaluated in 17 randomized trials assessed the role of radiotherapy after breast preservation surgery, demonstrating a reduction in the risk of 10-year recurrence from 35% to 19.3%, with no major differences in mortality reduction percent between pN0 or pN1 patients [50]. For patients with T3N0 breast cancer, evidence for the benefit and usefulness of post-mastectomy radiotherapy (PMRT) it is still controversial. Analyzing data from 13,901 patients with a median follow-up of 47 months, Almahariq and collaborators concluded that the benefit of PMRT is limited to patients who will not receive adjuvant chemotherapy. In cases of patients treated with neo-adjuvant chemotherapy (NAC), the addition of PMRT did not bring any benefit [51].

The chest wall irradiation technique includes two tangential fields, the total dose for standard regimen being 50 Gy in 25 fractions, 1 fraction per day, 5 days per week. The associated late toxicities include the increased cardiovascular risk, especially in association of radiotherapy with anthracycline-based chemotherapy or anti-HER2 target agents, but also pulmonary and cutaneous fibrosis. Regarding the risk of irradiation of the thyroid gland, special mention should be made for adjuvant irradiation of regional lymph nodes (LN) in the case of N + patients. The significant benefit of nodal levels irradiation is demonstrated especially for more than three LN +. Irradiation of the internal mammary nodes is controversial, being associated with a severe increase in the risk of radiation-induced cardiac toxicity. The technique of irradiation of the supraclavicular and infra-clavicular LN has evolved considerably by delineation of target volumes and OARs. Using advanced image guided radiation therapy (IGRT) protocol, the dose received by the thyroid gland can be limited. Currently, through the widespread use of the 3D-CRT technique and even IMRT/VMAT irradiation techniques, a more precise irradiation of all target volumes is possible [24,52]. Anatomical limits for all axillary and supraclavicular LN levels have been proposed for in order to create a consensus in delimitation for target volumes and OARs. Analyzing the anatomical limits of levels III and IV, we notice that the nodal volume in breast cancer radiotherapy is located in the immediate vicinity of the thyroid gland. As Li and collaborators remark, there is great variability in target volumes and OARs delineation between different radiotherapy centers. The implementation for breast cancer irradiation using IMRT/VMAT techniques based on image guided radiotherapy (IGRT) concept make it necessary to apply consensus in contouring in order to obtain the maximum therapeutic benefit, protecting OARs, including thyroid gland from a potential late toxicity [24,53,54].

Radiotherapy-induced hypothyroidism has been evaluated in breast cancer patients over the past decade, but the number of studies which analyze this subject is still low. A 21% rate of hypothyroidism in patients who received supraclavicular irradiation for breast cancer was reported by Akyurek et al. at a median interval of 9 months after completion of the treatment [55]. Bruning and collaborators reported a higher rate of hypothyroidism in supraclavicular irradiated patients [56]. The lowest rate of hypothyroidism at 2 years after treatment for patients with supraclavicular LN irradiation was reported by Wolny-Rokicka (6%), and thyroid gland function evaluation included TSH, free thyroxine (FT4), and free tri-iodothyronine (FT3) levels (Table 1) [57].

A study that included 4073 cases of breast cancer treated with adjuvant radiotherapy assessed the risk of radio-induced hypothyroidism in 3 different patient lots depending of the irradiated anatomical region (whole breast alone, regional nodal irradiation with cranial border subclavian artery and nodal irradiation including the supraclavicular lymph nodes with the cranial margin of the cricoid cartilage as delineation limit). The authors evaluated the mean dose received by the thyroid gland using dose conversion to uniform equivalent dose (EQD2) based on a linear-quadratic model (LQ), in order to estimate all toxicities in relation to standard fractionation. With a median follow-up of 84 months, an increased rate of hypothyroidism in patients who received radiotherapy in the supraclavicular region, the rates of hypothyroidism at 3 years being 0.8%, 0.9% and 2.2% for the 3 groups of patients evaluated. Significant differences were also identified from a dosimetric point of view between the 3 groups of patients, mean dose received by the thyroid being 0.23, 1.93, and 7.89 Gy, respectively. The study concludes the need to delineate and evaluate the radiation doses received by the thyroid gland in breast cancer [58]. The increased frequency for the use of new radiotherapy techniques with modulated intensity made it necessary the comparative evaluation of the doses received by the thyroid gland by conventional and three-dimensional irradiation techniques and by new radiotherapy techniques, taking into account the particularities of IMRT and VMAT techniques regarding the unpredictable dose distribution. Using computer tomography-based treatment plans from 10 breast cancer patients, initially treated with opposites tangential field-in-field to the chest wall and anteroposterior opposite radiation fields to the ipsilateral regional lymph nodes, Haciislamoglu and collaborators proposed radiotherapy alternative treatment plans by IMRT or helical tomotherapy technique (HT) in case the dosimetric constraints for the thyroid were applied or not. V30 < 50% and mean thyroid dose (Dmean) <21 Gy have been proposed as dose constraints. Dmean values of 30.56 + −5.38 Gy and median V30 = 55% obtained by opposites tangential field-in-field were higher than those obtained by IMRT and HT technique (if dose constraints were applied). The reduction of Dmean and V30 was significant for both IMRT and HT techniques, with Dmean being reduced from 25.56 Gy + −6.66 Gy and 27.48 + −4.16 Gy to 18. 57 + −2.14 and 17.34 + −2.70, respectively. The use of dosimetric constraints reduced V30 obtained by IMRT and HT technique from 33% and 36% to 18% and 17%, respectively. The study clearly demonstrates the need to use dosimetric constraints when reverse planimetry techniques are proposed [59]. Using national cancer registries, a Danish study assessed the risk of hypothyroidism in breast cancer survivors aged ≥35 years diagnosed with non-metastatic disease between 1996 and 2009. The study excluded women with pre-existing thyroid disease and included cases treated with chemotherapy and radiotherapy for both the breast and chest wall and for cases that required regional lymph node irradiation. The identification of the cases was based on the use of diagnostic codes as well as on the prescription of levothyroxine. The study included 44,574 cases evaluated compared to 203,306 patients in the control group. The 5-year cumulative incidence of hypothyroidism in breast cancer patients was significantly higher than that reported in the control group (1.8% vs. 1.6%). The study also shows an increased rate of hypothyroidism in patients receiving radiotherapy and chemotherapy compared to those who received surgery alone, mentioning the combination of chemotherapy and radiation therapy on the lymph nodes as factors that need to be known as risk factors for hypothyroidism [60]. The Dutch Society of Radiation Oncology breast cancer platform proposed establishing a consensus for the delimitation of target volumes and OARs based on cases proposed for planning in 19 main radiotherapy centers. It should be noted that the thyroid and more precisely v30 was rarely delimited as an OAR respectively evaluated. The consensus proposed the delimitation of thyroid thought and the evaluation of v30 only if nodal levels 3 and 4 are irradiated but did not propose a certain dose restriction [61]. A study published in 2008 by Smith and colleagues failed to demonstrate the role of radiotherapy and especially supraclavicular irradiation in elderly patients with stage <IV breast cancer. In a cohort of 38,255 aged >65 years compared with a control group of 111,944 cancer free patients, the incidence of hypothyroidism at 5 years was 14%. Considering 4 + positive lymph nodes as surrogate for supraclavicular irradiation and 0 positive lymph nodes as surrogates for omission of supraclavicular irradiation, the study demonstrates an increased incidence of hypothyroidism in elderly surviving breast cancer patients without being able to correlate it with supraclavicular irradiation [62]. A systematic review and meta-analysis that included five studies (478 cases) identified from 7 international databases and 3 national databases identified an increased rate of hypothyroidism (higher than other thyroid gland dysfunctions) and an increase in TSH values after radiation therapy in patients with breast cancer. Darvish and collaborators propose as a strategy to limit the toxic effects of irradiation to protect the thyroid gland, considering necessary dosimetric constraints and follow-up of patients after treatment [63].

A radiobiological model based on normal tissue complication probability (NTCP) proposed by Huang and collaborators aims to identify predictors of hypothyroidism in breast cancer patients receiving radiotherapy at the supraclavicular nodal level. The model was created using 192 cases, including both dosimetric data obtained from volume dose histograms (DVH) and clinical data. Subsequently, all collected data were correlated with the onset of clinical hypothyroidism. At a median interval of 25 months from completing the radiation treatment, 19.3% of patients receiving radiotherapy in the supraclavicular region developed hypothyroidism. Long thyroid glands and large volumes that received less than 20 Gy (CV20 Gy) have been identified as predictors of toxicity. The risk of hypothyroidism (<15%) was associated with CV20 > 8.5 cc and for small thyroid glands, while the mean dose and organ volume were correlated with the risk of hypothyroidism. A single institution dosimetric study conducted in India identified an average thyroid gland volume of 7.4 cc, lower than the values reported in the literature, considering that a thyroid volume of <8 cc is associated with an increased risk of hypothyroidism in patients with breast cancer. The authors also recommend monitoring thyroid function but also routine contouring of the thyroid gland as an OAR in all cases of breast cancer that will receive radiation therapy [8,64].

## 5. Conclusions

Hypothyroidism induced by LN irradiation in breast cancer treatment is an underestimated toxicity and increased survival rates even in the advanced or metastatic stages make it necessary to evaluate thyroid function after a complex multidisciplinary approach including supraclavicular radiotherapy. If the IMRT or VMAT irradiation technique is used, it is mandatory to delineate the thyroid gland as OAR and to apply dosimetric constraints in order to avoid its irradiation with high doses with potential risk of late toxicity. It is also necessary to assess the additional risk of hypothyroidism associated with new systemic oncological therapies that have significantly improved the prognosis.

## Figures and Tables

**Table 1:** Anatomical limits for delineation of level III (infraclavicular) and IV (supraclavicular) breast cancer radiotherapy [53,54].

Limits	Level III (Infraclavicular)	Level IV (Supraclavicular)
Superior	Superior margin of clavicle	5 mm superior of subclavian vein
Inferior	5 mm inferior to the subclavian vein	subclavian vein with a 5 mm margin
Medial	inside the pectoralis minor, level II and the Rotter level	jugular vein without margins, exclude common carotid artery and thyroide gland
Lateral	Clavicle and the jonction between infraclavicular and internal jugular vein	the tissue in proximity of external margin of scalene muscle and the anterior scalene muscles
Anterior	Posterior margin of pectoralis major	posterior part of the clavicle of the sternocleidomastoid and sterno-thyroid muscle
Posterior	Anterior from costae and Intercostals muscle	Pleura

## References

[1] Pernas S., Tolaney S.M., Winer E.P. (2018). CDK4/6 inhibition in breast cancer: Current practice and future directions. Ther. Adv. Med. Oncol..

[2] Agrawal S. (2014). Late effects of cancer treatment in breast cancer survivors. South Asian J. Cancer.

[3] Hancock S.L., McDougall I., Constine L.S. (1995). Thyroid abnormalities after therapeutic external radiation. Int. J. Radiat. Oncol..

[4] Jereczek-Fossa B.A., Alterio D., Jassem J., Gibelli B., Tradati N., Orecchia R. (2004). Radiotherapy-induced thyroid disorders. Cancer Treat. Rev..

[5] Bhandare N., Kennedy L., Malyapa R.S., Morris C.G., Mendenhall W.M. (2007). Primary and Central Hypothyroidism after Radiotherapy for Head-and-Neck Tumors. Int. J. Radiat. Oncol..

[6] Boomsma M.J., Bijl H.P., Langendijk J.A. (2011). Radiation-induced hypothyroidism in head and neck cancer patients: A systematic review. Radiother. Oncol..

[7] Jefferies W.M. (1961). Occult hypothyroidism and “metabolic insufficiency”. J. Chronic Dis..

[8] Huang H., Roberson J., Hou W., Mani K., Valentine E., Ryu S., Stessin A. (2020). NTCP model for hypothyroidism after supraclavicular-directed radiation therapy for breast cancer. Radiother. Oncol..

[9] Cella L., Conson M., Caterino M., De Rosa N., Liuzzi R., Picardi M., Grimaldi F., Solla R., Farella A., Salvatore M. (2012). Thyroid V30 Predicts Radiation-Induced Hypothyroidism in Patients Treated With Sequential Chemo-Radiotherapy for Hodgkin’s Lymphoma. Int. J. Radiat. Oncol..

[10] Norris A.A., Amdur R.J., Morris C.G., Mendenhall W.M. (2006). Hypothyroidism When the Thyroid Is Included Only in the Low Neck Field during Head and Neck Radiotherapy. Am. J. Clin. Oncol..

[11] Mercado G., Adelstein D.J., Saxton J.P., Secic M., Larto M.A., Lavertu P. (2001). Hypothyroidism: A frequent event after radiotherapy and after radiotherapy with chemotherapy for patients with head and neck carcinoma. Cancer.

[12] Lin C.-L., Wu S.-Y., Huang W.-T., Feng Y.-H., Yiu C.-Y., Chiang W.-F., Ho S.-Y., Lin S.-H. (2019). Subsequent thyroid disorders associated with treatment strategy in head and neck cancer patients: A nationwide cohort study. BMC Cancer.

[13] Sinard R.J., Tobin E.J., Mazzaferri E.L., Hodgson S.E., Young N.C., Kunz A.L., Malhotra P.S., Fritz M.A., Schuller D.E. (2000). Hypothyroidism After Treatment for Nonthyroid Head and Neck Cancer. Arch. Otolaryngol.-Head Neck Surg..

[14] Illés A., Bíró E., Miltényi Z., Keresztes K., Váróczy L., András C., Sipka S., Bakó G. (2002). Hypothyroidism and Thyroiditis after Therapy for Hodgkin’s Disease. Acta Haematol..

[15] Metzger M.L., Hudson M.M., Somes G.W., Shorr R.I., Li C.-S., Krasin M.J., Shelso J., Pui C.-H., Howard S.C. (2006). White Race As a Risk Factor for Hypothyroidism After Treatment for Pediatric Hodgkin’s Lymphoma. J. Clin. Oncol..

[16] Sinnott B., Ron E., Schneider A.B. (2010). Exposing the Thyroid to Radiation: A Review of Its Current Extent, Risks, and Implications. Endocr. Rev..

[17] PDQ Pediatric Treatment Editorial tabkleBoard (2021). Late Effects of Treatment for Childhood Cancer (PDQ^®^): Health Professional Version. PDQ Cancer Information Summaries [Internet].

[18] Chow E.J., Friedman D.L., Stovall M., Yasui Y., Whitton J.A., Robison L.L., Sklar C.A. (2009). Risk of thyroid dysfunction and subsequent thyroid cancer among survivors of acute lymphoblastic leukemia: A report from the Childhood Cancer Survivor Study. Pediatr. Blood Cancer.

[19] Tell R., Sjödin H., Lundell G., Lewin F., Lewensohn R. (1997). Hypothyroidism after external radiotherapy for head and neck cancer. Int. J. Radiat. Oncol..

[20] Oudin C., Auquier P., Bertrand Y., Chastagner P., Kanold J., Poirée M., Thouvenin S., Ducassou S., Plantaz D., Tabone M.D. (2016). Late thyroid complications in survivors of childhood acute leukemia. An L.E.A. study. Haematologica.

[21] Posner M.R., Ervin T.J., Fabian R.L., Weichselbaum R.R., Miller D., Norris C.M., Rose C. (1984). Incidence of hypothyroidism following multimodality treatment for advanced squamous cell cancer of the head and neck. Laryngoscope.

[22] Bentzen S.M., Constine L.S., Deasy J., Eisbruch A., Jackson A., Marks L.B., Haken R.T., Yorke E.D. (2010). Quantitative Analyses of Normal Tissue Effects in the Clinic (QUANTEC): An Introduction to the Scientific Issues. Int. J. Radiat. Oncol..

[23] Pacelli R., Caroprese M., Palma G., Oliviero C., Clemente S., Cella L., Conson M. (2019). Technological evolution of radiation treatment: Implications for clinical applications. Semin. Oncol..

[24] Barrett A., Morris S., Dobbs J., Roques T. (2009). Practical Radiotherapy Planning.

[25] Brodin N., Tomé W.A. (2018). Revisiting the dose constraints for head and neck OARs in the current era of IMRT. Oral Oncol..

[26] Kim M.Y., Yu T., Wu H.-G. (2014). Dose-volumetric Parameters for Predicting Hypothyroidism after Radiotherapy for Head and Neck Cancer. Jpn. J. Clin. Oncol..

[27] Akgun Z., Atasoy B.M., Ozen Z., Yavuz D., Gulluoglu B., Sengoz M., Abacioglu U. (2014). V30 as a predictor for radiation-induced hypothyroidism: A dosimetric analysis in patients who received radiotherapy to the neck. Radiat Oncol..

[28] Fujiwara M., Kamikonya N., Odawara S., Suzuki H., Niwa Y., Takada Y., Doi H., Terada T., Uwa N., Sagawa K. (2015). The threshold of hypothyroidism after radiation therapy for head and neck cancer: A retrospective analysis of 116 cases. J. Radiat. Res..

[29] Huang C., Tan H., Guo R., Zhang Y., Peng H., Peng L., Lin A., Mao Y., Sun Y., Ma J. (2019). Thyroid dose-volume thresholds for the risk of radiation-related hypothyroidism in nasopharyngeal carcinoma treated with intensity-modulated radiotherapy—A single-institution study. Cancer Med..

[30] American Cancer Society (2005). Cancer Facts and Figures 2005.

[31] Harris J.R., Lippman M.E., Morrow M., Osborne C.K. (2004). Diseases of the Breast.

[32] D’Eredita G., Giardina C., Martellotta M., Natale T., Ferrarese F. (2001). Prognostic factors in breast cancer: The predictive value of the Nottingham Prognostic Index in patients with a long-term follow-up that were treated in a single institution. Eur. J. Cancer.

[33] Olivotto I.A., Chua B., Sharon J.A., Caroline H.S., Chia S., Ragaz J. (2003). Long-term survival of patients with supraclavicular metastases at diagnosis of breast cancer. J Clin. Oncol..

[34] Chan C.W.H., Law B.M.H., So W.K.W., Chow K.M., Waye M.M.Y. (2017). Novel Strategies on Personalized Medicine for Breast Cancer Treatment: An Update. Int. J. Mol. Sci..

[35] Li L., Jiang G., Chen Q., Zheng J.N. (2014). Ki67 is a promising molecular target in the diagnosis of cancer (Review). Mol. Med. Rep..

[36] Joo W.D., Visintin I., Mor G. (2013). Targeted cancer therapy—Are the days of systemic chemotherapy numbered?. Maturitas.

[37] Jorns J.M. (2019). Breast Cancer Biomarkers: Challenges in Routine Estrogen Receptor, Progesterone Receptor, and HER2/neu Evaluation. Arch. Pathol. Lab. Med..

[38] Fujita N., Enomoto Y., Inakami K., Yanagisawa T., Iguchi C., Aono T., Nomura T., Yamamoto H., Kasugai T., Shiba E. (2019). Prognostic Factors in HER2-Positive Primary Breast Cancer Patients Treated Using Neoadjuvant Chemotherapy Plus Trastuzumab. Oncology.

[39] Honkanen T.J., Tikkanen A., Karihtala P., Mäkinen M., Väyrynen J.P., Koivunen J.P. (2019). Prognostic and predictive role of tumour -associated macrophages in HER2 positive breast cancer. Sci. Rep..

[40] Del Prete S., Montella L., Arpino G., Buono G., Buonerba C., Dolce P., Fiorentino O., Aliberti M., Febbraro A., Savastano C. (2020). Second line trastuzumab emtansine following horizontal dual blockade in a real-life setting. Oncotarget.

[41] Keith K.C., Lee Y., Ewend M.G., Zagar T.M., Anders C.K. (2016). Activity of transtuzumab-emtansine (TDM1) in HER2-positive breast cancer brain metastases: A case series. Cancer Treat Commun..

[42] Hurvitz S.A., Im S., Lu Y., Colleoni M., Franke F.A., Bardia A., Harbeck N., Chow L., Sohn J., Lee K.S. Phase III MONALEESA-7 trial of premenopausal patients with HR+/HER2− advanced breast cancer treated with endocrine therapy ± ribociclib: Overall survival results. Proceedings of the 2019 ASCO Annual Meeting.

[43] Sledge G.W., Toi M., Neven P., Sohn J., Inoue K., Pivot X., Burdaeva O., Okera M., Masuda N., Kaufman P.A. (2020). The effect of abemaciclib plus fulvestrant on overall survival in hormone receptor-positive, ERBB2-negative breast cancer that progressed on endocrine therapy–MONARCH 2: A randomized clinical trial. JAMA Oncol..

[44] Anker G.B., Lonning P.E., Aakvaag A., Lien E.A., Anker P.E.L.G.B. (1998). Thyroid function in postmenopausal breast cancer patients treated with tamoxifen. Scand. J. Clin. Lab. Investig..

[45] Gordon D., Beastall G.H., McArdle C.S., Thomson J.A. (1986). The effect of tamoxifen therapy on thyroid function tests. Cancer.

[46] Mazokopakis E.E., Karefilakis C.M., Tsartsalis A.N., Milkas A.N., Starakis I.K. (2008). Exemestane-Induced Subclinical Hypothyroidism. Clin. Drug Investig..

[47] Hamnvik O.P., Larsen P.R., Marqusee E. (2011). Thyroid dysfunction from antineoplastic agents. J. Natl. Cancer Inst..

[48] Rivas A.M., Larumbe-Zabala E., Diaz-Trastoy O., Schurr R.N., Jones C., Abdulrahman R., Dar N., Lado-Abeal J. (2020). Effect of chemoradiation on the size of the thyroid gland. Bayl. Univ. Med Cent. Proc..

[49] De Groot S., Janssen L.G.M., Charehbili A., Dijkgraaf E.M., Smit V.T.H.B.M., Kessels L.W., Van Bochove A., Van Laarhoven H.W.M., Kranenbarg E.M.-K., Van Leeuwen-Stok A.E. (2015). Thyroid function alters during neoadjuvant chemotherapy in breast cancer patients: Results from the NEOZOTAC trial (BOOG 2010-01). Breast Cancer Res. Treat..

[50] Early Breast Cancer Trialists’ Collaborative Group (EBCTCG) (2011). Effect of radiotherapy after breast-conserving surgery on 10-year recurrence and 15-year breast cancer death: Meta-analysis of individual patient data for 10 801 women in 17 randomised trials. Lancet.

[51] Almahariq M.F., Quinn T.J., Siddiqui Z.A., Thompson A.B., Jawad M.S., Chen P.Y., Gustafson G.S., Dilworth J.T. (2020). Post-mastectomy radiotherapy is associated with improved overall survival in T3N0 patients who do not receive chemotherapy. Radiother. Oncol..

[52] Li X.A., Tai A., Arthur D.W., Buchholz T.A., Macdonald S., Marks L.B., Moran J.M., Pierce L.J., Rabinovitch R., Taghian A. (2009). Variability of target and normal structure delineation for breast-cancer radiotherapy: A RTOG multi-institutional and multi-observer study. Int. J. Radiat. Oncol. Biol. Phys..

[53] Offersen B.V., Boersma L.J., Kirkove C., Hol S., Aznar M.C., Sola A.B., Kirova Y.M., Pignol J., Remouchamps V., Verhoeven K. (2016). ESTRO consensus guideline on target volume delineation for elective radiation therapy of early stage breast cancer, version 1.1. Radiother Oncol..

[54] Gee H.E., Moses L., Stuart K., Nahar N., Tiver K., Wang T., Ward R., Ahern V. (2018). Contouring consensus guidelines in breast cancer radiotherapy: Comparison and systematic review of patterns of failure. J. Med. Imaging Radiat. Oncol..

[55] Akurek S., Babalioglu I., Kose K., Gokce S.C. (2014). Thyroid Dysfunction Following Supraclavicular Irradiation in the Management of Carcinoma of the Breast. Int. J. Hematol. Oncol..

[56] Bruning. P., Bonfrèr J., De Jong-Bakker M., Nooyen W., Burgers M. (1985). Primary hypothyroidism in breast cancer patients with irradiated supraclavicular lymph nodes. Br. J. Cancer.

[57] Wolny-Rokicka E., Tukiendorf A., Wydmański J., Roszkowska D., Staniul B.S., Zembroń-Łacny A. (2016). Thyroid Function after Postoperative Radiation Therapy in Patients with Breast Cancer. Asian Pac. J. Cancer Prev..

[58] Choi S.H., Chang J.S., Byun H.K., Son N.-H., Hong C.-S., Hong N., Park M.Y.-I., Kim J., Kim J.S., Kim Y.B. (2021). Risk of Hypothyroidism in Women After Radiation Therapy for Breast Cancer. Int. J. Radiat. Oncol..

[59] Haciislamoglu E., Canyilmaz E., Gedik S., Aynaci O., Serdar L., Yoney A. (2019). Effect of dose constraint on the thyroid gland during locoregional intensity-modulated radiotherapy in breast cancer patients. J. Appl. Clin. Med. Phys..

[60] Falstie-Jensen A.M., Esen B., Kjærsgaard A., Lorenzen E.L., Jensen J.D., Reinertsen K.V., Dekkers O.M., Ewertz M., Cronin-Fenton D.P. (2020). Incidence of hypothyroidism after treatment for breast cancer—A Danish matched cohort study. Breast Cancer Res..

[61] Hurkmans C., Duisters C., Peters-Verhoeven M., Boersma L., Verhoeven K., Bijker N., Crama K., Nuver T., van der Sangen M. (2021). Harmonization of breast cancer radiotherapy treatment planning in the Netherlands. Tech. Innov. Patient Support Radiat. Oncol..

[62] Smith G.L., Smith B.D., Giordano S.H., Shih Y.C.T., Woodward W.A., Strom E.A., Perkins G.H., Tereffe W., Yu T.K., Buchholz T.A. (2008). Risk of hypothyroidism in older breast cancer patients treated with radiation. Cancer.

[63] Darvish L., Ghorbani M., Teshnizi S.H., Roozbeh N., Seif F., Bayatiani M.R., Knaup C., Amraee A. (2018). Evaluation of thyroid gland as an organ at risk after breast cancer radiotherapy: A systematic review and meta-analysis. Clin. Transl. Oncol..

[64] Madisetty A., Suryadevara A., Chinta S.K., Vuppu S., Marella V.R.R. (2020). Should we consider thyroid gland as an organ at risk in carcinoma breast patients receiving adjuvant radiation by conformal technique? A single institute dosimetric study. Indian J. Cancer.

